# Rapamycin Attenuates Endothelial Apoptosis Induced by Low Shear Stress via mTOR and Sestrin1 Related Redox Regulation

**DOI:** 10.1155/2014/769608

**Published:** 2014-01-22

**Authors:** Junxia Zhang, Zhimei Wang, Junjie Zhang, Guangfeng Zuo, Bing Li, Wenxing Mao, Shaoliang Chen

**Affiliations:** Department of Cardiology, Nanjing First Hospital Affiliated to Nanjing Medical University, Changle Road 68, Nanjing, Jiangsu 210006, China

## Abstract

*Background*. Studies indicate the dramatic reduction of shear stress (SS) within the rapamycin eluting stent (RES) segment of coronary arteries. It remains unclear about the role of rapamycin in endothelialization of stented arteries where SS becomes low. Since mTOR (mammalian target of rapamycin) pathway is involved in the antioxidative sestrins expression, we hypothesized that rapamycin attenuated low SS (LSS) induced endothelial dysfunction through mTOR and sestrin1 associated redox regulation. *Methods and Results*. To mimic the effect of LSS on the stented arteries, a parallel plate flow chamber was used to observe the interplay of LSS and rapamycin on endothelial cells (ECs). The results showed LSS significantly induced EC apoptosis which was mitigated by pretreatment of rapamycin. Rapamycin attenuated LSS induced reactive oxygen species (ROS) and reactive nitrogen species (RNS) production via prohibition of sestrin1 downregulation. Activities of mTORC1 and mTORC2 were detected contradictorily modulated by LSS. Inhibition of rictor expression by target small interfering RNA (siRNA) transfection prohibited sestrin1 downregulation induced by LSS, but inhibition of raptor did not. *Conclusions*. Rapamycin may prohibit sestrin1 downregulation through targeting mTORC2 in appeasing LSS induced EC oxidative apoptosis. Our results provide the *in vitro* evidence to explain the pathophysiology of RES stented arteries.

## 1. Introduction

Rapamycin, the specific inhibitor of mTOR (mammalian target of rapamycin), is a natural product of the soil actinomycete streptomyces hygroscopicus [1]. Rapamycin and its derivatives are widely employed in the rapamycin eluting stent (RES) because they successfully prevent in-stent restenosis [2]. However, rapamycin is suspected to compromise endothelial function and induce endothelial apoptosis [3] and even implicate to delayed endothelialization [4–7]. Conversely, other researchers found rapamycin was not such cytotoxic to ECs as it arrested ECs at G0/G1 phase of cell cycle without inducing apoptosis [8]. Furthermore, rapamycin was reported to attenuate high-amplitude, mechanical stretch-induced apoptosis in pulmonary microvascular endothelial cells [9]. Therefore, the potential of rapamycin on EC apoptosis needs to be clarified.

Low shear stress (LSS) is a well-established risk factor resulting in endothelial dysfunction and atherosclerotic lesions [10, 11]. Data from our previous *in vivo* studies demonstrated that SS on the luminal surface of stented coronary artery reduced dramatically after implantation of RES [12, 13]. In the parallel flow chamber study we found that LSS induced endothelial apoptosis and accumulation of reactive oxygen species (ROS) [14]. However, the role of rapamycin on endothelial oxidative stress and apoptosis induced by LSS remains unknown.

Sestrins are a small gene family which encode three conserved proteins in mammals, sestrin1, sestrin2, and sestrin3 [15]. Sestrins, which exhibit oxidoreductase activity, are transcripted by p53 and FoxO to increase antioxidative responses upon stress [16–18]. Sestrins are on the convergence of oxidative insults and the mTOR signals [19–21]; therefore, we hypothesized rapamycin attenuated endothelial oxidative stress and apoptosis induced by LSS via mTOR and sestrins related redox regulation.

## 2. Materials and Methods

### 2.1. Cell Culture

Human umbilical vein endothelial cells (HUVECs) were obtained from Cellbank of Chinese Academy of Sciences (Shanghai, China). Rat aortic endothelial cells (RAECs) were isolated from male Sprague-Dawley rats (Laboratory Animal Center of Nanjing Medical University, Nanjing, China) according to the method described previously [22]. The use of animal material in this study conforms to the International Guiding Principles for Biomedical Research Involving Animals. All animal handling procedures were approved by the animal ethics board of Nanjing Medical University. Cells were cultured in DMEM (GIBICO) supplemented with 10% fetal bovine serum (GIBICO), at 37°C in a 5% CO_2_ incubator. Passage 5–8 of RAECs was used in this research.

### 2.2. Application of LSS

The parallel flow chamber was made by Shanghai Medical Instrument School (Shanghai, China) as described [23]. In brief, by sandwiching a silicon gasket between two stainless steel plates, the cells grown to confluence on coverslip were on the lower plate and subjected to fluid flow powered by a reciprocal pump. SS of 2 dyne/cm^2^ on the parallel surface of the plate can be obtained by modulating the proportion of fluid volume passing the flow chamber to that shunting into tank.

### 2.3. Materials and Reagents

Rapamycin, DAPI, 4,5-Diaminofluorescein diacetate (DAF-2DA), and dihydroethidium (DHE) were purchased from Sigma-Aldrich. MitoSOX Red, MitoTracker Red CMX ROS, and Trizol reagents were obtained from Invitrogen. Primary antibodies of phospho-mTOR (Ser2448), mTOR, phospho-p70 S6 (Thr389), p70 S6 Kinase, phospho-Akt (Ser473), Akt, Rictor, Raptor, and secondary antibody were obtained from Cell Signaling Technology. Reverse transcription reagent Kit and SYBR real-time PCR kit were from Takara (Dalian, China). Terminal deoxynucleotidyl transferase-mediated nick end labeling (TUNEL) kit was purchased from Roche Applied Science (Indianapolis, IN).

### 2.4. Detection of Apoptosis

The cells were treated with DAPI dye at a final concentration of 10 *μ*g/mL for 10 min before microscopic observation. The DNA strand breaks were detected using a TUNEL kit according to the manufacturer's instructions.

### 2.5. Western Blotting

Protein samples containing 40 *μ*g total protein were separated on 10% SDS-PAGE gels and transferred to PVDF membranes. The membranes were incubated with antibodies and visualized by chemiluminescence. The intensity of bands was quantified by NIH Image J software 1.43.

### 2.6. Detection of Reactive Oxygen Species (ROS) and Reactive Nitrogen Species (RNS)

For detection of RNS and ROS, cells were gently washed twice with PBS and incubated in HBSS solution containing 5 *μ*mol/L DAF-2DA for 30 min, 5 *μ*mol/L DHE for 20 minutes, 2 *μ*mol/L mitoTracker for 15 min, and 5 *μ*mol/L mitoSOX for 10 min at 37°C. Images were obtained by fluorescence microscope and imported into Image J software where the fluorescent densities and sizes were analyzed under fixed thresholds.

### 2.7. siRNA

Rictor and raptor small interfering RNA (siRNA) and control siRNA were purchased from Dharmacon. Transfection of ECs with siRNA (100 nM) was performed using Hiperfect (Qiagen) according to the manufacturer's instructions. In brief, subconfluent HUVECs were grown on coverslip in serum free medium with siRNAs. After 24 h of transfection, the cells were washed one time and cultured in medium with 10% FBS. Western blot was performed to confirm the efficiency of siRNA knockdown.

### 2.8. Real-Time PCR

Nuclear extracts were prepared using Trizol reagent and quantified using a Nanodrop 2000. The sequences of the forward and reverse strands for human sestrin1 primers used were forward: 5′-GCATGTTCCAACATTTCGTG-3′ and reverse: 5′-GTTCCAAATTGCCCGTCTAA-3′. For human gapdh, the primer sequences were 5′-TGAGAAGTATGACAACAGCCTCA-3′ and 5′-AGTCCTTCCACGATACCAAAGTT-3′. Messenger RNA levels of sestrin1 gene relative to reference gapdh were determined by two-step real-time PCR.

### 2.9. Statistics

The data were expressed as the mean ± standard deviation. The comparison of groups was performed by student *t* test or one-way ANOVA analysis. The Newman Keuls test was applied for post hoc pairwise multiple comparisons. A level of *P* < 0.05 was considered significant. One sample *t* test was used for the comparison of mRNA expression by relative quantitative RT-PCR, where concentration of control group was deemed as a constant 1. 95% confidence interval not including 1 was considered significant.

## 3. Results

### 3.1. Rapamycin Mitigated LSS Induced Endothelial Apoptosis

**Figure 1:**
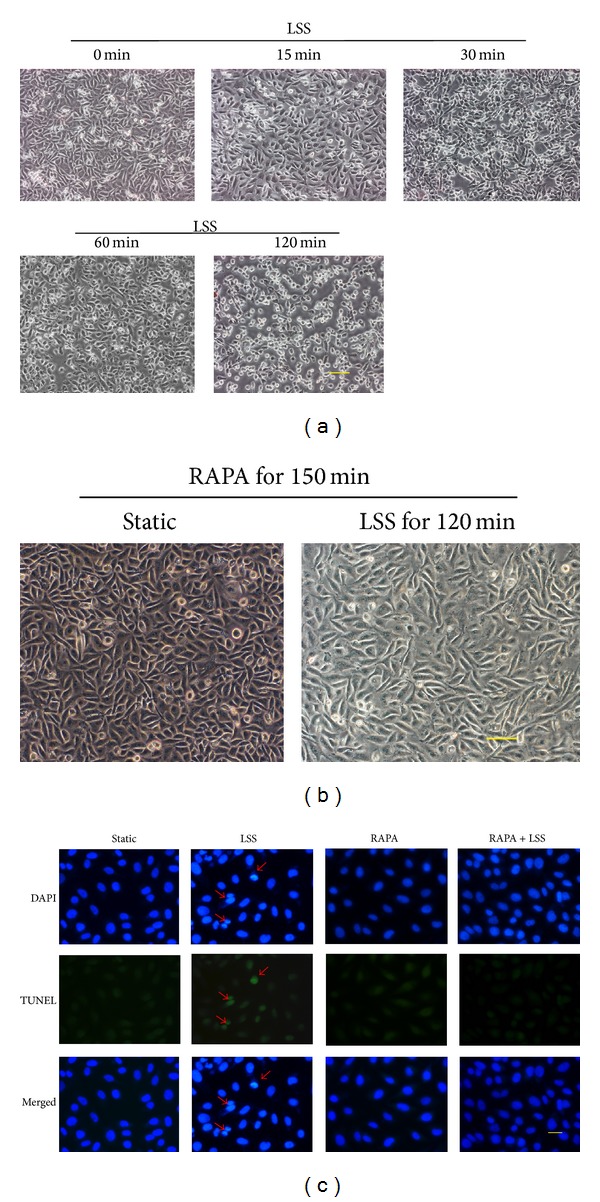
LSS induced EC apoptosis was attenuated by rapamycin. LSS at 2 dyne/cm^2^ caused EC viability reduction and apoptosis which was attenuated by rapamycin (RAPA) at the concentration of 100 ng/mL (a) HUVECs shape change and detachment after being subjected to LSS for the various time points. (b) Pretreated HUVECs with RAPA for 30 min and continuously delivered RAPA during flow study for 120 min abrogated LSS effect on cell shape change and detachment. (c) DAPI and TUNEL staining were used to label apoptotic RAECs (arrows show apoptotic cells). Adding RAPA appeased LSS induced apoptosis and RAPA alone had no influence on apoptosis. Scale bars: 100 *μ*m ((a)-(b)), 25 *μ*m (c).

LSS caused significant decrease in HUVECs viability with cell shrinkage and easy detachment from the coverslip compared to static culture (Figure 1(a)). Rapamycin with the concentration of 100 ng/mL was delivered 30 min before application of LSS and continuously used in flow medium for 120 min. Rapamycin preserved HUVECs shape and viability after exposure to LSS while rapamycin alone had no impact on morphology and viability of HUVECs at static condition (Figure 1(b)). After exposure to flow, apoptotic RAECs stained by DAPI increased in contrast to those in static state and this trend was ameliorated by rapamycin. Apoptotic cells were not increased by treatment of rapamycin at static condition. TUNEL assay further confirmed effect of LSS on EC apoptosis and the protective effect of rapamycin (Figure 1(c)).

### 3.2. Rapamycin Attenuated LSS Induced ROS/RNS Production in HUVECs

**Figure 2:**
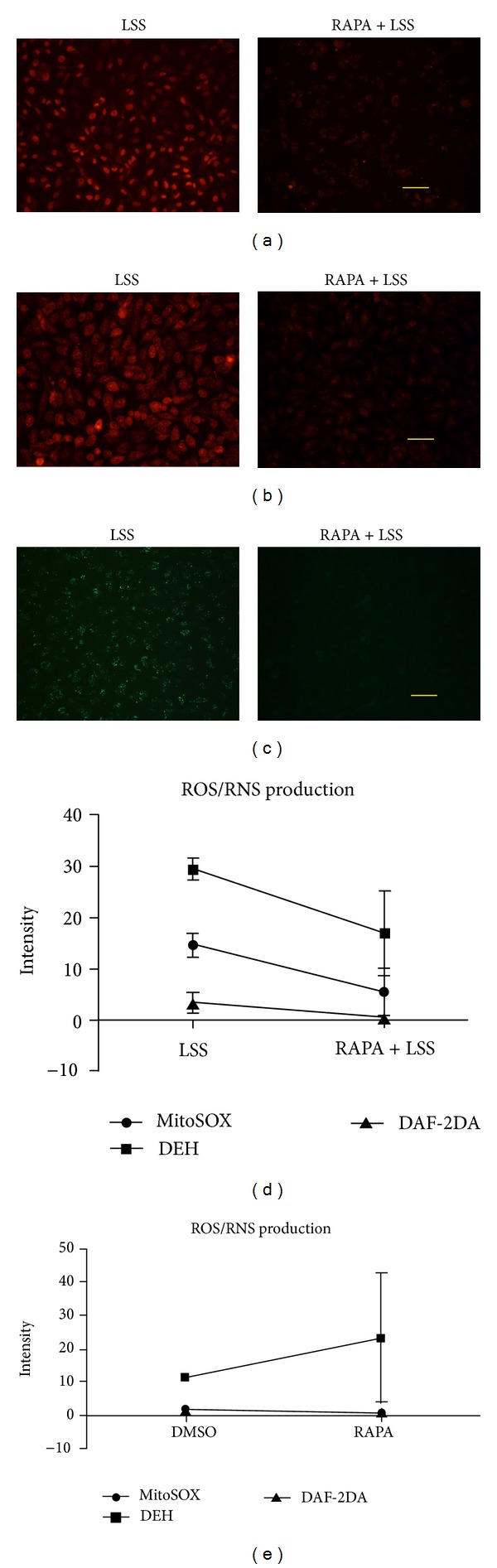
Rapamycin attenuated LSS induced ROS/RNS production in HUVECs. ROS/RNS accumulation caused by LSS at 2 dyne/cm^2^ for 120 min was attenuated by rapamycin (RAPA) at the concentration of 100 ng/mL in HUVECs. (a) RAPA ameliorated the effect of LSS on promoting ROS production examined by mitoSOX. (b) Consistent result was observed with DHE staining. (c) RAPA attenuated LSS induced RNS accumulation assayed by DAF-2DA. Scale bars: 50 *μ*m ((a)–(c)). (d) The line graph was the statistical results of fluorescence intensity and size at the fixed color threshold which showed the significant reduction of ROS or RNS production in RAPA + LSS group, *P < 0.05; *n* = 10. (e) RAPA did not significantly increase ROS or RNS production as compared with vehicle DMSO, *P* = 0.1279 with ANOVA, *n* = 10.

Rapamycin ameliorated the effect of LSS on promoting HUVECs mitochondrial ROS production assayed by mitoSOX (Figure 2(a)). Consistent result was observed with DHE in HUVECs (Figure 2(b)). Rapamycin attenuated LSS induced RNS accumulation detected by DAF-2DA (Figure 2(c)), which was a probe to detect peroxynitrite in the presence of superoxide [24]. The comparison of fluorescence intensity demonstrated that rapamycin could reduce ROS/RNS production induced by LSS (Figure 2(d)). Rapamycin alone had no effect on ROS/RNS production (Figure 2(e)).

### 3.3. Protection of Rapamycin from LSS Induced ROS/RNS in RAECs

**Figure 3:**
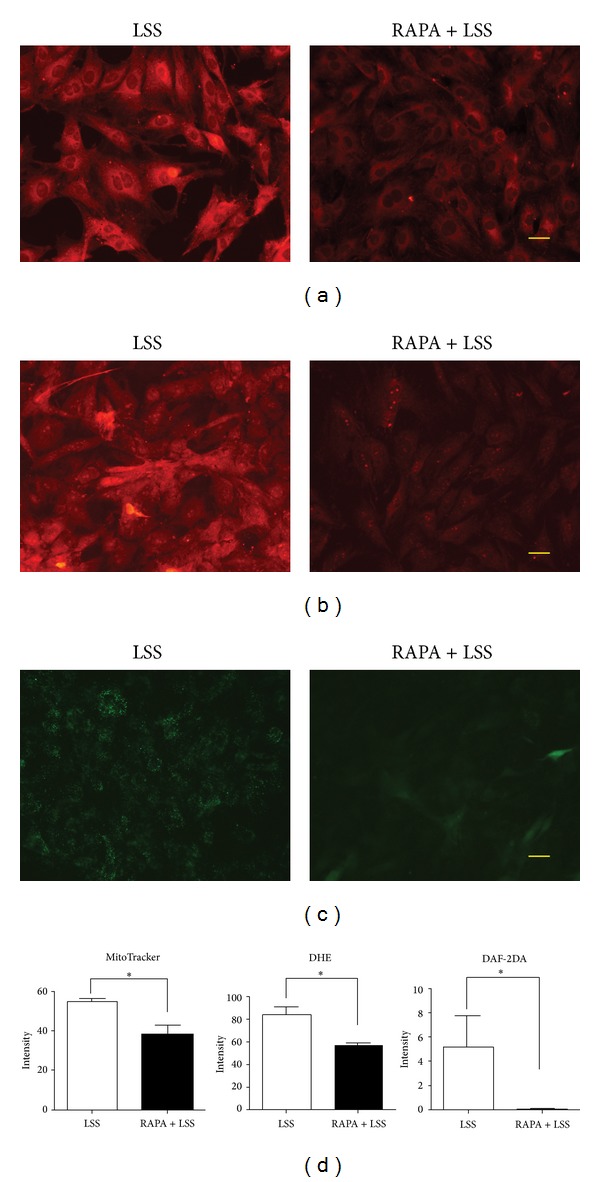
Rapamycin attenuated LSS induced ROS/RNS production in RAECs. ROS/RNS accumulation caused by LSS at 2 dyne/cm^2^ was attenuated by rapamycin (RAPA) at the concentration of 100 ng/mL in RAECs. (a) RAPA ameliorated the effect of LSS on promoting ROS production examined by mitoTracker. (b) Consistent result was observed with DHE staining. (c) Rapamycin attenuated LSS induced RNS accumulation assayed by DAF-2DA. Scale bars: 25 *μ*m ((a)–(c)). (d) The column graphs demonstrated the fluorescence intensity and size was significantly reduced in RAPA + LSS group as compared with LSS group, *P* < 0.05, *n* = 4.

The effect of rapamycin on appeasing LSS induced mitochondrial ROS production was confirmed in RAECs assayed by mitoTracker (Figure 3(a)). Protection of rapamycin was testified with DHE in RAECs (Figure 3(b)). Rapamycin attenuated LSS induced RNS accumulation detected by DAF-2DA was also reaffirmed in RAECs (Figure 3(c)). The quantified analysis of fluorescence intensity was demonstrated in column graphs (Figure 3(d)).

### 3.4. LSS Reduced Sestrin1 Expression and Caused Contradictory Activation of mTORC1 and mTORC2

**Figure 4:**
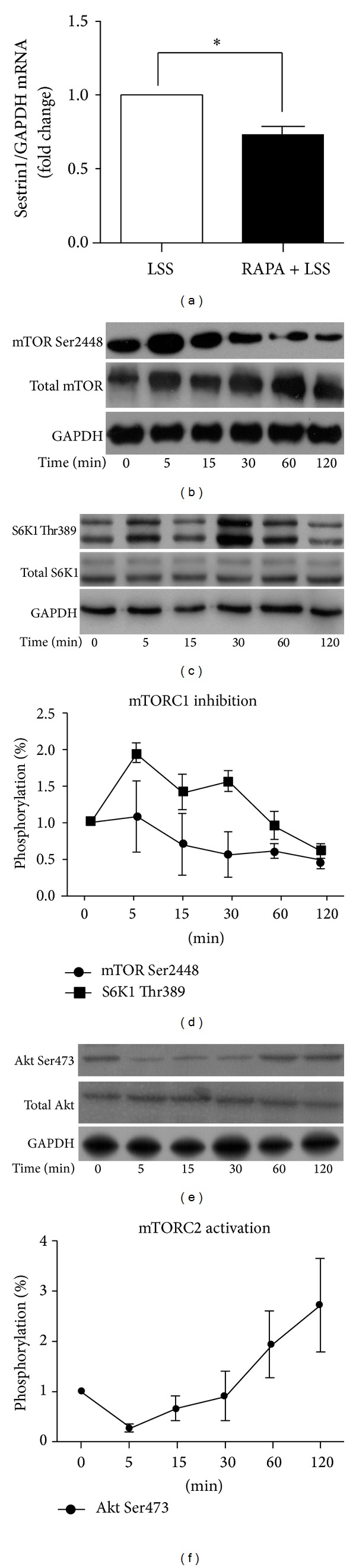
LSS reduced sestrin1 expression and caused contradictory activation of mTORC1 and mTORC2 in HUVECs. LSS reduced sestrin1 expression and caused contradictory activation of mTORC1 and mTORC2 in HUVECs. (a) Gene expression of sestrin1 normalized to the house-keeping gene gapdh was significantly decreased after shearing HUVECs at 2 dyne/cm^2^ for 120 min compared to static cells,*P < 0.05; *n* = 4. (b) Immunoblotting of mTORC1 activity at mTOR Ser2448 and S6K1 Thr389 phosphorylation sites (c). (d) The line graph below indicated the ratio of phosphorylated kinases to total proteins for each shearing time, with that of static cells set as 0 min. All data are from 3 independent experiments. (e) mTORC2 activity was determined by immunoblotting of Akt Ser473 phosphorylation with densimetric analysis of 3 independent experiments (f).

To study the mechanism of rapamycin on mitigation of LSS induced oxidative apoptosis, antioxidative sestrin1 gene expression and the activities of two complexes of mTOR, mTORC1 and mTORC2, were measured after shearing HUVECs. Sestrin1 transcription decreased after shearing HUVECs at 2 dyne/cm^2^ for 120 min as compared with static cells (Figure 4(a)). To test the mTORC1 activity, mTOR phosphorylation at Ser2448 site and its downstream S6K1 Thr389 phosphorylation were examined. After application of LSS, the phosphorylation of mTOR at Ser2448 site in HUVECs was maximally activated at 5 min and declined after 15 min (Figure 4(b)). The phosphorylation for S6K1 in HUVECs was also gradually inhibited by LSS after a transient activation (Figure 4(c)). The downward trend of the two kinases' activity was incorporated in the line graph where percentage of phosphorylated kinases was quantified relative to the total kinases (Figure 4(d)). The phosphorylation of Akt Ser 473 which served as readout of mTORC2 activation was increased by LSS (Figure 4(e)). The upward trend was demonstrated in the line graph (Figure 4(f)).

### 3.5. Prohibition of Sestrin1 Downregulation by Rapamycin Sheltered ECs from LSS Insult via mTORC2 Inhibition

**Figure 5:**
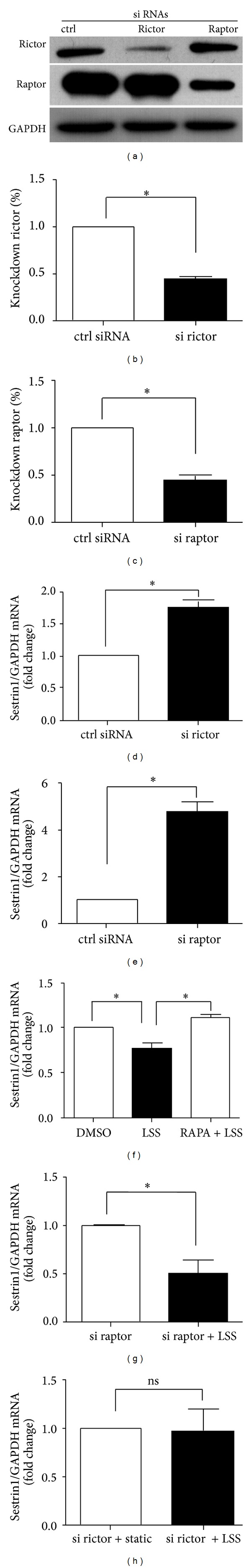
Upregulation of sestrin1 expression by rapamycin protected ECs from LSS insults via mTORC2 inhibition. Upregulation of sestrin1 expression by rapamycin protected ECs from LSS insults via mTORC2 inhibition. (a) HUVECs were treated with siRNA against rictor, raptor, or scrambled control siRNA (ctrl siRNA). 48 h after transfection, cells were harvested for the detection of Rictor, Raptor, and GAPDH protein levels by western blot. ((b)-(c)) Percentage of knockdown rictor or raptor was analyzed relative to GAPDH, *P < 0.05; *n* = 3. ((d)-(e)) 48 h after transfection, sestrin1 expression was significantly elevated in rictor siRNA or raptor siRNA group, *P < 0.05; *n* = 4. (f) Rapamycin (RAPA) reduced LSS induced sestrin1 decreasing,*P < 0.05, *n* = 4. (g) After mTORC1 inhibition with raptor siRNA transfection for 48 h, HUVECs were exposed to LSS or kept static. Expression of sestrin1 determined by real time PCR was reduced after LSS exposure, *P < 0.05, *n* = 4. (h) After inhibition of mTORC2 with rictor siRNA, sestrin1 expression was not decreased by LSS in HUVECs as compared with static group, *P* = 0.7731, *n* = 4.

Inhibition of rictor and raptor expression by small interfering RNAs (siRNAs) was used to prevent mTORC2 and mTORC1 assembly and thus to inhibit their functions (Figure 5(a)). Column graph showed the effectiveness of knockdown by siRNAs (Figures 5(b)-5(c)). Both inhibition of mTORC2 and mTORC1 assembly increased sestrin1 expression (Figures 5(d)-5(e)). Rapamycin at the concentration of 100 ng/mL diminished LSS induced sestrin1 reduction in HUVECs (Figure 5(f)). Inhibition of mTORC1 by raptor siRNA could not prohibit the decreasing of sestrin1 expression when exposed to LSS (Figure 5(g)). However, mTORC2 inhibition by rictor siRNA could prohibit the reduction of sestrin1 expression induced by LSS (Figure 5(h)).

## 4. Discussion

The main finding of the current study is that rapamycin protects ECs from LSS induced oxidative apoptosis by prohibiting antioxidative sestrin1 gene downregulation via mTORC2 inhibition.

The implication of SS changes on RES implantation is yet lack of mechanistic explanation [25]. After implanting bare metal stent, SS relates inversely to the intimal thickness because LSS induces vascular smooth muscle cells (SMCs) proliferation [26] and high SS induces SMCs apoptosis [27]. However, our previous hemodynamic studies and that of others [28] found SS maintained to be low after deployment of RES because rapamycin abrogated LSS effect on intimal proliferation. In light of these observations, we speculate that LSS maintained by RES and rapamycin itself interplay in the pathophysiology of stented segment of coronary artery and participate in the delayed endothelialization of stent surface.

LSS was reported to initiate endothelial apoptosis [29]. An *in vivo* study uncovered that apoptosis of the ECs in the vessel wall was characterized in the downstream of plaques where LSS occurred [30], similar to our previous studies [14, 31]. The current study provided the evidence that when rapamycin was delivered 30 min in advance and continuously used in flow medium, endothelial apoptosis induced by LSS could be ameliorated.

It was demonstrated that rapamycin induced vascular dysfunction by increasing superoxide production and decreasing nitric oxide (NO) synthesis [32]. Nonetheless, another group reported that rapamycin appeased oxidative stress with the consequence of attenuating senescent endothelial dysfunction [33]. It is plausible because aging is a disease of oxidative stress and rapamycin is a medication to prolong life span [34, 35]. In corneal ECs, rapamycin at 25 and 50 nM of concentration reduced tert-butyl hydroperoxide induced apoptosis [36]. Rapamycin was also reported to protect vasculature by preserving nitric oxide (NO) mediated vascular reactivity [37] and by inhibiting hydrogen peroxide induced vascular loss of contractility [38] and even reduced endothelial apoptosis confronting mechanic stress [9]. These results are consistent with our data that rapamycin protects ECs from oxidative apoptosis induced by LSS.

mTOR functions in the formation of two complexes, mTORC1 and mTORC2. Blocking complexes assembly can inhibit mTOR activity. mTORC1, the mTOR-raptor complex, is sensitive to rapamycin. mTORC2 that contains rictor can be inhibited by rapamycin in ECs in prolonged time course [39]. Biophysical cues were reported to activate cellular mTOR pathway recently [40]. Time course study indicated mTORC1 was inhibited by LSS after a transient activation while mTORC2 was activated. The contradictory activity of mTORC2 and mTORC1 possibly made mTORC2 to be the priority candidate inhibited by rapamycin other than mTORC1. Cheng et al. [41] reported rapamycin modulated eNOS expression in high and low SS conditions, implying that mTOR was activated in regulation of eNOS expression when exposed to both high and low SS. Furthermore, they found that the modulation was discrete and that rapamycin reduced high SS boosted eNOS expression while it attenuated LSS reduced eNOS expression. Nonetheless, they failed to propose the rationale of the contradictory modulation of eNOS by rapamycin in the setting of SS. Provided LSS activated the same mTOR complex as high SS, rapamycin should have the consistent other than discrete effect on eNOS expression in both high and low SS exposure. Through specific inhibition of mTORC1 and mTORC2 by siRNAs, this present study uncovered that mTORC2, the insensitive action site of rapamycin, was the actual target of rapamycin in LSS exposed ECs.

This study also found antioxidative sestrin1 was downregulated in LSS exposed ECs, implying LSS induced ROS/RNS accumulation could partly be due to the diminished sestrin1 expression. Considering there is no report about the posttranscription regulation of sestrins expression, this study next investigated sestrin1 gene expression by inhibition of mTORC1 and mTORC2. Sestrin1 expression was boosted by inhibition of mTORC1 and mTORC2. In the presence of LSS, we further identified the inhibiting site of rapamycin was mTORC2 in sheared ECs. Upregulation of sestrin1 by rosiglitazone reduced ROS and protected retinal cells against apoptosis [42], congruous to this study that boosting of sestrin1 expression by rapamycin mediated redox dependent antiapoptotic effect.

In the current study we selected 2 dyne/cm^2^ as the LSS applied on ECs since 0 to 4 dyne/cm^2^ was usually used as LSS in the parallel flow chamber research. This was obviously different from SS mapped on the reconstructed lumen surface of stented coronary arteries, on which LSS was defined as less than 12 dyne/cm^2^ [43] or even higher [13]. Therefore, results from the present study must be interpreted cautiously considering the different conditions *in vitro* and *in vivo*.

In conclusion, our results fit well into a model that rapamycin alleviates LSS induced oxidative apoptosis by prohibiting sestrin1 downregulation through mTORC2 inhibition. Our results provide an *in vitro* evidence to explain the pathophysiology of RES stented segments of coronary arteries.
